# Compensation for Patients with Work-Related Lung Cancers: Value of Specialised Occupational Disease Consultations to Reduce Under-Recognition

**DOI:** 10.3390/ijerph22060927

**Published:** 2025-06-12

**Authors:** Clémence Roux, Mélanie Fafin-Lefevre, Rémy Morello, Laurent Boullard, Bénédicte Clin

**Affiliations:** 1 Occupational Health Department, Caen University Hospital, 14000 Caen, France; cle96@hotmail.fr; 2 UFR Santé, Caen Normandie University, 14000 Caen, France; melanie.fafin-lefevre@unicaen.fr (M.F.-L.); morello-r@chu-caen.fr (R.M.); 3 Biostatistics and Clinical Research Unit, Caen University Hospital, 14000 Caen, France; 4 PST14, 14000 Caen, France; lboullard@pst14.fr; 5 INSERM U1086 «ANTICIPE», 14000 Caen, France

**Keywords:** cancer, lung, occupational exposure, work-related cancer, occupational disease, compensation, recognition

## Abstract

Purpose: The aim of this retrospective study was to analyse the compensation procedures concerning patients presenting with work-related lung cancer (LC), hospitalised in a French university hospital, and to assess the benefit of systematic specialised occupational disease (OD) consultations in improving procedures for reporting and recognising OD. Methods: Patient exposure to occupational lung carcinogens was assessed via an analysis of a standardised questionnaire, completed between 1 January 2009 and 24 April 2023. Among the 2024 patients who completed the questionnaire, 621 patients with probable exposure to occupational lung carcinogens were included. Among these patients, two groups were compiled: group 1, consisting of the 392 subjects who did not benefit from specialised OD consultations, and group 2, consisting of the 229 subjects who benefited from such consultations since 2014 and to whom a medical certificate to claim for compensation was issued by a physician. During the second phase of our study, we determined the outcome of the compensation procedure for OD. Uni- and multivariate logistic regressions were performed according to descending logistic regression methods. Results: Multivariate analyses, including smoking status, sex, age and claim for compensation, confirm the significant relationship between specialised OD consultation and claim for compensation (OR 18.13, 95% CI [11.47–28.64]). Furthermore, the rate of occupational disease recognition has multiplied by 1.5 since 2014. Conclusion: This study confirms the importance of specialised OD consultations in helping patients with LC to obtain compensation and to reduce under-recognition.

## 1. Introduction

Lung cancer (LC) is the most common cancer worldwide (the most common in men and the second most common in women), with 2.48 million new cases in 2022, and is the leading cause of death from cancer, with over 1.8 million deaths in 2022 [1]. In France, with approximately 33,438 new cases in 2023, LC is the second most common cancer in men after prostate cancer and the leading cause of death from cancer (with 22,800 deaths annually). In women, the incidence of LC and is associated mortality are increasing (19,339 new cases in 2023 and 10,300 deaths by LC in 2018), and is the second leading cause of death after breast cancer [2].

Worldwide, 78% of male LC cases and 53% of female LC cases are attributed to tobacco consumption [3]. However, many occupational carcinogens have been identified by the International Agency for Research on Cancer (IARC) as risk factors for LC. This is the most common cancer localisation associated with occupational origin [4,5]. Identifying potential occupational exposure to carcinogens is associated with major medical and social challenges. All countries should maintain a registration system to provide information to policymakers on the incidence and prevalence of occupational diseases (OD), based on the International Labour Organization Convention concerning Occupational Safety and Health and the Work Environment [6].

In Europe, between 2013 and 2022, 37,022 cases of occupational cancer, including 15,272 cases of LC, often linked to asbestos exposure, were officially recognised as OD. The most common types of occupational cancers were LC and mesothelioma (with 14, 914 cases) combined, representing 81.5% of all newly reported occupational cancer cases during this period [7].

In France, it is estimated that between 2000 and 6000 cases of LC per year are attributable to occupational exposure [5,8]. Even if the rate of medicolegal compensation for occupational cancers has increased by a factor of three in 24 years, in France, under 2000 cancers are compensated (or ‘recognised’) as OD each year. Indeed, in 2022, 1652 cases of cancer were compensated (recognised) as ODs, 60.5% of which were LC, 92% of LC being related to asbestos exposure [9,10].

As in France, the under-reporting of occupational cancers is also noted by many other industrialised countries, despite the existence of mandatory reporting laws. However, identifying cases of occupational cancer is a genuine collective challenge in order to improve understanding and to ensure effective prevention. It is also an individual challenge for victims, since, in the case of OD recognition, compensation is generally higher than that provided by disease and disability insurance. Also, several studies have been conducted worldwide to improve the identification of occupational carcinogenic exposure, by means of occupational questionnaires [11,12]. Several studies in France have contributed to improving the identification of occupational carcinogenic exposure with the aim of increasing the reporting and recognition of LC as OD. In these studies, a standardised questionnaire was issued to patients presenting with LC upon the request of a specialised OD practitioner, to identify potentially attributable occupational exposure by tracing the patient’s entire career [13]. Other studies evaluated the value of systematically setting up an OD consultation when issuing an AQREP self-questionnaire (Auto-Questionnaire for occupational exposure) [14,15,16,17]. Other projects to improve the reporting of OD have been initiated in various countries, essentially for specific categories of OD [18].

In the university hospital where our study was conducted, systematic occupational questionnaires for patients with LC treated in the pulmonary and cardiothoracic surgery departments were implemented in 2007. Prior to 2014, after an analysis of the questionnaire by a specialised OD physician, a letter was addressed to the general practitioner of any patient considered to be exposed to carcinogens, outlining the conclusions of the questionnaire analysis with regard to the patient’s eligibility to claim for compensation. As of 2014, consultations in the specialised OD department were systematically proposed to patients considered as exposed to lung carcinogens.

Our analysis aimed to determine the most frequently observed lung carcinogen, the recommended procedures for OD recognition and the outcome of formalities enabling patients to claim for the recognition of occupational LC.

The main objective of the study was to analyse the value of specialised OD consultations in patients considered to be exposed after questionnaire analysis. The further aim was to determine their relevance and effectiveness in improving procedures for reporting and recognising OD.

## 2. Literature Review

### 2.1. Lung Cancer Due to Occupational Exposures

Occupational cancers are the result of exposure to carcinogenic agents in the workplace. Occupational exposure to various agents has been linked to an increased risk of lung cancer [19]. Indeed, several carcinogenic agents and exposure situations have been classified as definite carcinogens by the International Agency for Research on Cancer (IARC) (group 1), with an increased incidence of lung cancer: asbestos, arsenic and arsenic compounds, benzo(a)pyrene, beryllium and beryllium compounds, bis(chloromethyl) ether and chloromethyl methyl ether, cadmium and cadmium compounds, hexavalent chromium derivatives, diesel engine emissions, sulphur mustard, coal tar, coal tar pitch, soot, coal gasification and coke production, work in iron and steel foundries, certain nickel derivatives, plutonium-239, radon-222, X-rays and gamma rays and daughter products (work in iron ore mines), crystalline silica, the painting profession, passively inhaled tobacco smoke, talc containing asbestiform fibres, aluminium production using the Söderberg process, the rubber industry and welding fumes. Occupational carcinogens are responsible for approximately 15% of LC cases each year in France [5,8].

The International Labour Organization (ILO) estimates that 666,000 deaths worldwide are attributable to occupational cancer annually, which is double the number of fatalities from occupational accidents [20,21]. According to the Global Burden of Disease, Injury and Risk Factors (GBD) study, occupational exposure to 14 carcinogens is believed to be responsible for approximately 350,000 cancer deaths [22,23]. In the European Union, occupational cancer accounts for an estimated 102,500 deaths each year, twenty times the number of deaths resulting from occupational accidents. Lung cancer alone constitutes a substantial proportion, ranging from 54% to 75% of all occupational cancers. Epidemiological studies further suggest that occupational exposures are responsible for 5.3% to 8.4% of all cancers, and a striking 17% to 29% of all lung cancer deaths among men [7,24].

### 2.2. Compensation Measures Provided in France and Across the Globe for Patients Presenting with LC

As previously mentioned, identifying potential occupational exposure to carcinogens is associated with major medical and social challenges. Western European countries have compiled lists (or tables, which is the case in France and Italy) of ODs entitling victims to compensation, including occupational cancer. These lists do not, however, have the same value from one country to another, conferring a more or less firm presumption of professional ‘imputability’ [25,26]. The recognition of cancer as an OD can be provided when, after investigation, the national insurance organisation validates the presence of all medical and legal requirements; however, such requirements differ from one country to another [25,26,27]. In the USA, each state has its own workers’ compensation system covering OD, including cancer, provided there is a demonstrable link between the disease and workplace exposure. However, the specifics, such as the covered conditions, filing deadlines, and benefit calculations, differ state by state. Claimants must typically prove that their cancer is directly related to their job duties or exposures. This often involves medical evidence and, in some cases, expert testimony. Certain states have adopted ‘presumptive’ laws for specific occupations [28]. In France, the foundations of OD compensation are based on the law of 25 October 1919: an OD being defined as ‘the consequence of a worker’s exposure to a risk during their work and over a long period.*’* The advantage of the occupational risk hedging scheme is that the victim benefits from the ‘presumption of origin’ for injury sustained in the workplace, without having to prove any fault on the part of their employer. For the French general social security scheme, as for the ‘Régime Agricole’ (scheme specifically designed for agricultural workers), tables have been compiled, detailing, for each OD, the criteria for the origin of the listed diseases to be considered occupational. These tables allow the recognition of OD via ‘presumption of origin’: if the criteria on the table is satisfied, proof of the occupational origin is no longer required to be given. LC related to occupational carcinogens may be recognised as an OD under an existing table, or recognised as an ‘unlisted’ OD if there is no table for the specific carcinogen, yet a ‘direct and essential’ link is established between exposure and disease (the presumption of origin does not apply in the absence of an OD table) [25]. Once the occupational origin of the disease is recognised, affected individuals are entitled to several benefits: 100% coverage of medical expenses related to the occupational disease; higher daily allowance provided during periods of work stoppage compared to non-occupational illnesses; and a ‘Permanent Disability Annuity’, paid in cases of permanent sequelae or disability resulting from the disease. In the event of death, a lump sum death benefit and weekly payments for dependent children may be provided to the family. France has also established additional compensation mechanisms for asbestos victims, including the Fund for the Compensation of Asbestos Victims (FIVA), created in 2002, following a court judgment acknowledging state responsibility and the principle of social solidarity for asbestos-related health issues. Anticipated retirement provisions are also available for these patients. Such advantages justify the importance of establishing a possible relationship between the pathology presented and its professional origin, to ensure patient compensation for the associated loss [29].

### 2.3. Screening Procedures for Occupational Lung Cancer

The under-reporting of occupational cancers, particularly lung cancers, is a major obstacle to prevention. Lung cancer screening programmes have been established in several industrialised countries. The National Lung Screening Trial (NLST) in the United States [30] demonstrated the efficacy of chest CT screening, with repeated annual examinations in populations of smokers with a smoking history of over 30 pack-years, and in those who had stopped smoking less than 15 years prior to the trial. The NLST results, guidelines and expert opinions have been published all internationally [31,32,33,34,35].

In France, the screening of patients previously exposed to asbestos follows recommendations from the public hearing organised upon the initiative of the Haute Autorité de Santé, which recommends the use of a chest scanner [36]. However, in this case, the conditions for carrying out this examination are adapted to the screening of non-malignant pleuro-pulmonary lesions associated with asbestos exposure, and not to the operational screening of CBP. Recent confirmation of the effectiveness of such screening by chest CT was indeed based on a shorter timescale before repeating the examination (annual periodicity in practice) [30]. In addition, the relevance of this screening, demonstrated in a smoking population, should be evaluated in populations exposed to bronchopulmonary carcinogens in the workplace. Consequently, in France, monitoring procedures after occupational exposure to lung carcinogens are the subject of good medical practice recommendations, which call for experimentation aimed at validating the indication of the thoracic tomodensitometric tool in populations considered to be at high risk of LC [37]. Furthermore an ongoing feasibility study of low-dose chest CT lung cancer screening in subjects currently or previously occupationally exposed to lung carcinogens and at a high risk of lung cancer was implemented in 2018 in certain French regions [38].

## 3. Material and Methods

### 3.1. Study Design and Ethical Considerations

This retrospective study, conducted from 1 January 2009 to 24 April 2023, was approved by the Local Research Ethics Committee (Comité Local d’Ethique de la Recherche—CLER, n°4517). After analysis, the committee approved our study, indicating that it was deemed to be in compliance with the required ethical rules and standards. All participants received information on the study and gave their consent when they agreed to complete the questionnaire.

### 3.2. Study Population

The study population was recruited from the department of pneumology and cardiothoracic surgery in a French university hospital. Patients covered by the French general social security scheme presenting with primitive LC confirmed by histological diagnosis and who agreed to complete a questionnaire were included in the study.

Patients covered by the agricultural insurance scheme, patients with uncertain diagnosis, and patients presenting a different diagnosis from primitive LC were excluded.

Indeed, an analysis of claims for OD recognition for patients covered by the agricultural insurance scheme was not pertinent, since the tables for OD recognition are not comparable (differences in care pathway timescales or limited lists pertaining to professional tasks). Furthermore, certain tables in the general insurance scheme do not exist in the agricultural scheme.

### 3.3. Systematic Screening Procedure

Two research assistants from the university hospital went to the bedside of patients hospitalised for LC in the department of pneumology or cardiothoracic surgery, to conduct a face-to-face interview to complete the questionnaire.

The purpose of this questionnaire was to collect information on the patient’s work history (job title, start and end dates, employer, sector of activity and tasks performed for each profession), as well as self-reported occupational exposures.

Prior to 2014, for patients whose questionnaire identified a link between their cancer and previous occupational exposures, the occupational physician addressed a letter to the patient’s general practitioner to inform them of the patient’s eligibility to apply for OD recognition of their LC. For those whose questionnaire did not identify a link between cancer and possible occupational exposure, or for those whose exposure was not sufficiently characterised to allow OD recognition, the physician also addressed a letter to the general practitioner to inform them accordingly.

Thus, prior to 2014, a specialised OD consultation was not systematically proposed to these patients.

Since 2014, patients whose questionnaire identified possible or probable exposure in relation to their cancer were systematically given the opportunity to attend a specialised OD consultation. At the end of the consultation, a medical certificate was issued and information about applying for OD recognition explained to the patient when such recognition was likely. When sufficient exposure was not found after an analysis of the questionnaire, a letter was addressed to the patient’s general practitioner, as noted above.

**Figure 1:**
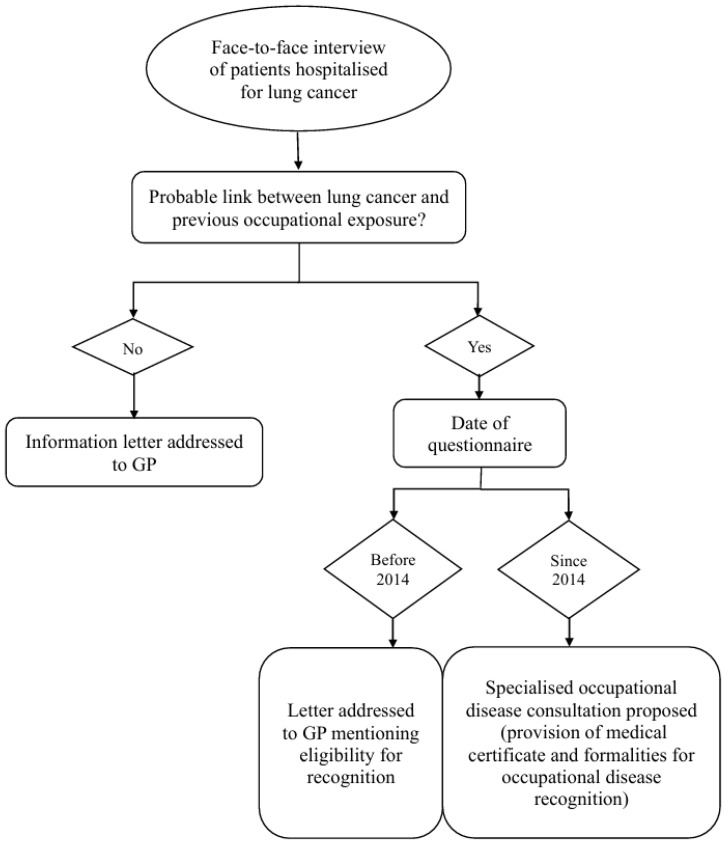
Systematic screening procedure flowchart.

Patients with a probable link between LC and previous occupational exposure were then separated into 2 groups for further analysis: one group consisting of patients having benefited from a specialised OD consultation and a second group that did not benefit from a specialised OD consultation. The different steps involved in the systematic screening procedure are summarised in the flowchart below (Figure 1).

### 3.4. Data Collection

The data collected for this study were:

- patient socio-demographic data (date of birth, sex)
- histological cancer type
- smoking status
- exposure to the most relevant lung carcinogen for the OD recognition process (since, in France, only one carcinogen can be retained for this procedure)
- recognition as an OD: on the grounds of an OD table for a given carcinogen, or on the grounds of an ‘unlisted’ disease in the absence of an OD table for the carcinogen in question

The outcome of the OD recognition claim for patients covered by the general social security insurance scheme was collected from CARSAT (Caisse d’Assurance Retraite et de la Santé au Travail) by providing the patient’s social security number. We were able to collect the administrative instruction date of the application, the year of OD recognition, the response to the claim (approved or rejected), and the occupational carcinogen concerned.

### 3.5. Statistical Analysis Method

For categorical variables, the differences between groups were tested using Pearson’s chi-squared test or, if the validity of the latter was not met, by the Fisher exact test. These statistical analyses were performed with BiostatTGV (a free biostatistics website that allows statistical tests online using R software). A two-sided *p* < 0.05 was considered to be statistically significant.

Uni- and multivariate logistic regressions predicting the probability of specialised consultation were performed according to descending logistic regression methods using IBM SPSS Statistics for Windows, Version 23.0. Armonk, NY, USA: IBM Corp. The selection of variables included in the model (smoking status, sex, age and claim for compensation) was based on a significance threshold equal to 0.20 in univariate analysis. The goodness-of-fit of the model was evaluated by the Hosmer–Lemeshow statistic. The Odds Ratio (OR) is provided with 95% Confidence Intervals (95CI).

## 4. Results

Among the 3006 eligible patients, 2024 completed the questionnaire between 1 January 2009 and 24 April 2023. Nine hundred and eighty-two patients refused to complete the questionnaire, indicating that they were not interested in possible procedures for OD recognition.

Of the 2024 patients who completed the questionnaire, 1229 (61%) had no exposure or their exposure was insufficiently characterised to be eligible for OD recognition, and 795 of the patients interviewed had possible or probable exposure to one or more carcinogenic agent(s) that could be linked to their LC. Among them, 174 patients, who did not meet the inclusion criteria, were excluded from the study (93 patients who had no histological diagnosis of primary LC, 3 patients covered by the agricultural insurance scheme, and 78 patients who either refused to be contacted after the questionnaire or who were since deceased).

Finally, 621 patients were included in the analysis; these patients had completed the questionnaire and were considered to be exposed to at least one occupational risk factor related to their LC after the questionnaire analysis. Only 11 consultations were conducted before 2014, and 218 were conducted after 2014.

Among these 621 patients, two groups were compiled:

- Group 1: 392 patients who did not benefit from a specialised OD consultation but for whom a letter was addressed to their general practitioner with advice on OD reporting;
- Group 2: 229 subjects who did benefit from a specialised OD consultation and for whom a medical certificate for an OD claim was provided at the end of the consultation.

**Figure 2:**
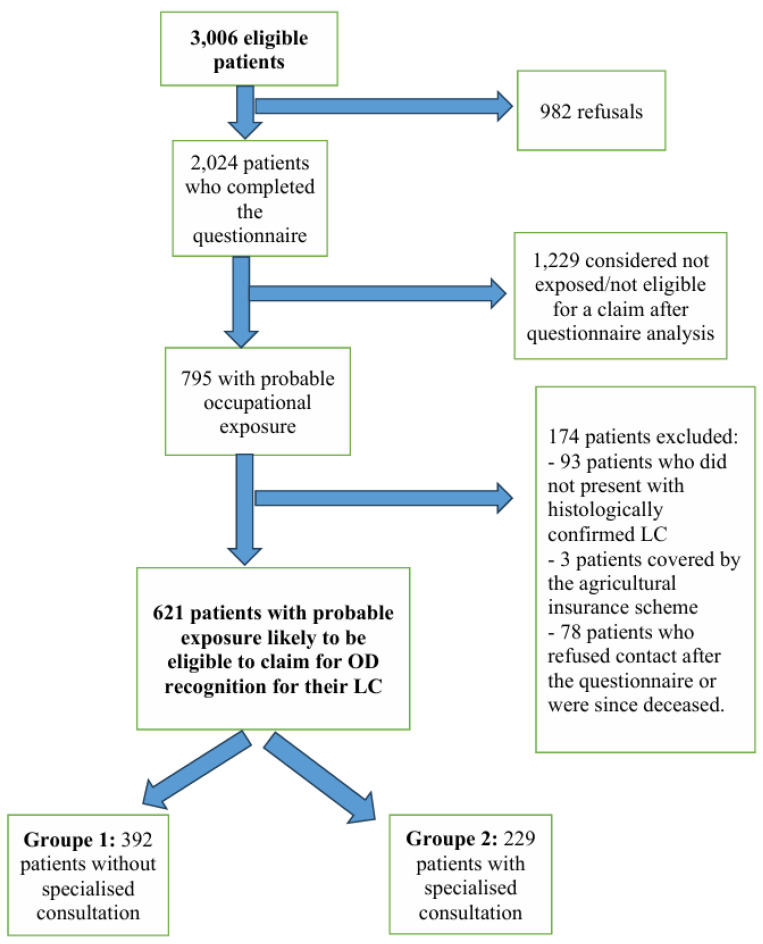
Study flowchart.

All these elements are summarised in the flowchart below (Figure 2).

The analysis covered 621 patients with LC and probable or sufficiently characterised exposure to at least one occupational carcinogen potentially related to their cancer, and patients who were eligible for OD recognition.

The characteristics of this population are described in Table 1.

The majority of these patients were men (97.9%), born between 1945 and 1965 (70.5%). The most frequent smoking status was ‘ex-smoker’ (66%). The majority of patients in the study had smoked over 30 pack-years. The most common histological type was pulmonary adenocarcinoma (52%). The most relevant occupational exposure for an OD claim found after the questionnaire analysis and during consultation was, by far, asbestos (84%), followed by crystalline silica (2.7%), welding fumes (2.7%), diesel engine fumes (2.6%) and the painting trade (2.4%) (Table 2).

As previously mentioned, among the 621 patients in our study, 392 (63%) only benefited from a letter addressed to their general practitioner recommending an OD recognition claim but did not benefit from a specialised OD consultation (group 1), and 37% were provided with a medical certificate issued by the occupational physician after a specialised consultation (group 2).

Patients who benefited from a specialised OD consultation after which they received a medical certificate (group 2) were significantly more likely to file an OD claim for their LC (*p* = 2,87.10^−48^). Indeed, in group 1, only 11.5% of subjects initiated any such claim, against 68.5% of subjects in group 2.

A comparison of groups 1 and 2 is presented in Table 2.

To confirm the effect of specialised consultations, uni- and multivariate logistic regressions were performed according to descending logistic regression methods (Table 3).

Multivariate analyses, including smoking status, sex, age and claim for recognition, confirm the significant relationship between specialised OD consultations and recognition claims (OR 18.13, 95% CI [11.47–28.64]) (Table 3).

In addition, of the 621 patients exposed from 2009 to 2023, 202 patients (32%) filed a claim for OD recognition, whether they benefited from an OD consultation or not. In order to determine whether there were differences between the subjects who had or had not claimed for OD recognition, regardless of the group, we compared the patients who had filed a claim with those who had not. The results of this comparison are shown in Table 4. The only significant difference was smoking status, with active smokers significantly less successful in their claims (*p* = 2,8.10^−5^).

From 2009 to 2023, of the 202 patients who filed an OD claim, 157 (78%) had benefited from a specialised OD consultation and had received a medical certificate, and 45 (22%) had not benefited from any such consultation or certificate (*p* < 0.01).

Concerning the patients who filed a claim before 2014, 16 (47%) obtained OD recognition, whereas 18 (53%) of claims were rejected. Since 2014, 121 (72%) patients obtained OD recognition, whereas 29 (17%) of claims were rejected (calculated *p*-value using Pearson’s chi-squared 4.9.10^−5^). The rate of OD recognition was therefore multiplied by 1.5 (72/47) since 2014.

Among the 137 patients who obtained OD recognition for their LC, 126 (92%) were associated with exposure to asbestos.

## 5. Discussion

Occupational lung cancer is a significant and often underestimated global public health issue, stemming from diverse workplace exposures to carcinogens. Despite mandatory reporting laws, the under-reporting of occupational cancers is noted in many industrialised countries, with cases of occupational lung cancer remaining largely under-reported and under-compensated worldwide [11,12,18,26]. This under-reporting is linked in particular to the high latency of LC, which makes it difficult to identify the risk factors involved, but also the fact that their possible occupational origin does not present any specific anatomo-clinical and/or prognostic character. It is therefore essential to identify the carcinogenic agents and occupational exposure situations involved in LC, in order to implement effective prevention strategies as well as to closely monitor potentially exposed employees.

Furthermore, for a patient presenting with LC, the identification of a relationship with their occupation should systematically be sought, in order to enable them to obtain compensation for their cancer through its recognition as an occupational disease. The International Labour Organization (ILO) plays a significant role in promoting international standards for occupational disease compensation. ILO Conventions [6] encourage member states to provide compensation for occupational diseases, including lung cancer caused by asbestos, and list various recognised occupational diseases.

The results of our study demonstrate that, since the implementation of systematic specialised OD consultations in 2014, the rate of claims filed by patients presenting with LC to obtain compensation for their cancer through recognition as an occupational disease has been multiplied by 6. In our study, the rate of OD recognition for patients having filed a claim was 68% (137/202). These results are comparable with those of a French study (58.4%) [17], the reference study conducted in Lyon (67%) [16] and the pilot study conducted by the Grenoble University Hospital (77%) [39]. In a study conducted in Italy [40], physicians from various departments referred all new cases of primary LC to an occupational physician. When occupational exposure to lung carcinogens was presumed, clinical reports were issued to the notifying physicians with advice on claims for medico-legal compensation. Prior to the implementation of the Italian study, few cases were referred to the occupational physician, and even fewer were compensated. An occupational aetiology was acknowledged in 182 (26%) of the 693 subjects referred for full evaluation. In another study [41], a long-running hospital-based programme invited all lung cancer patients for referral to an occupational health practitioner. Before the programme, only a few dozen cases had ever been evaluated, whereas, throughout the programme, 3274 lung cancer cases were referred and 1522 were fully interviewed. Occupational causation was identified in 395 patients (26% of those interviewed). All 395 were notified as potential occupational cancers (compared to the previous ‘handful’), 39% of them ultimately obtaining compensation. This systematic approach allowed the identification of hundreds of previously unrecognised occupational lung cancers, dramatically increasing OD claims.

In a retrospective study conducted in Romania [42], of 304 patients presenting with LC, 60 (20%) were referred for consultation at the OD centre and 27 were declared to have an OD.

Another study was conducted in Belgium in 2008 [11] to assess the number of cases of occupational LC in a Belgian cancer centre. From 1 September 2009 to 31 January 2011, each new patient with LC was referred for consultation to identify potential occupational exposure to lung carcinogens. Among 81 patients, 28 (35%) were found to have been definitely or probably exposed to one or more lung carcinogens (known or suspected) and thirteen OD compensation claims were filed (nine of which were approved, one rejected and three pending).

In North America, the formal screening of all lung cancer patients for occupational causes is rare, leading to severe under-recognition. This is underlined by compensation data: even in a highly industrialised province such as Ontario, only 1.2% of lung cancer deaths (and 0.6% nationally) were recognised as of occupational origin (2006–2010) [43], despite an estimated ~15% attributable fraction. The same study reported that compensated cases were almost all asbestos-related (lung cancers and mesotheliomas), meaning that other carcinogens remained largely unclaimed. The pattern is similar in the United States, with occupational lung cancers being ‘grossly neglected’ in routine practice and surveillance [44]. In a recent study [45] the authors linked data from the American College of Radiology’s Lung Cancer Screening Registry to ‘Medicare and Surveillance, Epidemiology, and End Results cancer registry’ data from 2015 to 2021. These data linkages provided an opportunity to better understand the early implementation of lung cancer screening in the United States. Studies from South Africa and multi-country African cohorts highlighted significant disparities in access to compensation for migrant workers, with systemic barriers such as documentation, cross-border legal issues, and lack of outreach [46].

In France, the process of compensation involves a detailed assessment of the individual’s occupational history and exposure to known carcinogens. However, the administrative procedures can be complex and time-consuming, often taking longer than the life expectancy of lung cancer patients. This complexity can deter certain patients from pursuing their claims [16,47].

Different hypotheses put forward in the literature may explain why claims are more likely to be filed after a specialised OD consultation. General practitioners do not always have the time to provide information on the required formalities for OD recognition when receiving the medical certificate and letter from the occupational health department, and they might consider such administrative procedures to be outside of their scope of action or care. Furthermore, in France, the context of medical demography concerning occupational physicians in particular is a matter of concern. Between 2006 and 2016, the number of occupational physicians decreased from 7000 to 5000, with a high average age. This could explain the decline in the number of medical certificates for OD drawn up by occupational physicians in France. In contrast, the time afforded for exchange between the occupational health practitioner and the patient during specialised OD consultations in the hospital is crucial for discussing the pertinence of claiming for OD recognition. Indeed, information is provided directly (with no intermediary) and the consultation offers an opportunity to answer the patient’s questions on exposure and to inform them about the stakes involved in medico-administrative compensation. For example, during the consultation, the practitioner drafts the medical certificate and partly completes the claim form for OD recognition (which must then be fully completed and signed by the patient) so as to help the patient as best they can throughout this formality. Thus, the OD consultation is perfectly relevant within the scope of medico-legal recognition. It is nevertheless important to note that, even with assistance from an experienced professional and the provision of all pertinent information, 32% of patients having benefited from a specialised consultation did not file a claim for OD recognition.

Data from the literature suggests that refusal to file a claim can be for a number of reasons. There may be a self-censure mechanism or a sense of illegitimacy among active smokers who may attribute the cause of their LC to tobacco consumption, hence the importance of adequately informing patients of the carcinogenic role of certain risk factors or of occupational exposure. Indeed, the high prevalence of tobacco consumption in patients presenting with LC has been identified as a barrier to the identification of occupational LC [48,49]. Completing the questionnaire or attending an OD consultation may be perceived as an extra ‘burden’ for patients who are already exhausted by their disease and its treatment [39]. Certain patients may also experience difficulties in completing the formalities involved in claiming for OD recognition of their cancer (administrative difficulties) [14,50,51].

We also chose to report the main carcinogenic agent to which patients were exposed and for which an OD recognition claim was recommended by the physician, given that, in France, any such recognition can only be obtained for a listed OD. In our study, we observed that asbestos is the most frequently involved carcinogen, since 126 patients were granted OD recognition associated with asbestos exposure; this was from a total of 137 recognised patients (i.e., 92%). This rate is comparable with data published in the 2022 report published by the French Medical Insurance scheme, which reported that the LC of 923 patients was associated with asbestos out of a total of 1000 patients recognised the same year [9]. Similarly, 94.8% of the patients included in a multicentric French study [17] obtained OD recognition associated with asbestos exposure.

This high rate can be explained by the fact that, when a patient is exposed to asbestos and to other pulmonary carcinogenic agents during their career, asbestos is the agent to which physicians grant priority for claiming OD recognition. Indeed, with specific regard to asbestos, concurrent to a possible claim for OD recognition, in France, the patient can claim for compensation from the FIVA (Compensation Fund for Asbestos Victims), the mission of which is to ‘fully compensate for damage to victims of asbestos.’.

Hence, a number of patients were exposed to several carcinogenic agents throughout their professional career, and the figures presented in our study underestimate their exposure to other carcinogenic agents for which a claim was not recommended. Certain agents that are carcinogenic for humans, listed in group 1 by the International Agency for Research on Cancer, are not currently listed in the OD tables. This is particularly the case for certain industrial processes (such as the painting trade, the rubber industry, diesel engine fumes and welding fumes). In this case, the French Occupational disease Recognition Committee (CRRMP) may be called upon to rule on an ‘unlisted’ disease, but it can be difficult to plausibly prove the direct and essential link between LC and occupational exposure, notably when extraprofessional risk factors such as smoking are present. For sixty patients (9.7%) in our study population, a claim for recognition of an ‘unlisted’ OD was recommended. Among these patients, eight filed a claim and three obtained OD recognition.

The demographic data for patients in our study who completed the questionnaire are similar to those in the literature on bronchopulmonary cancer, with regard to age, sex, smoking status and histology. The mean age of patients considered to be exposed at the time of questionnaire completion was 64 years. Hence, the majority of patients were either retired or approaching retirement. This can be explained by the higher proportion of male subjects exposed to carcinogenic agents [17].

Our study has certain limitations. Indeed, a selection bias could exist, since 982 patients refused to complete the questionnaire and, unfortunately, we did not have access to the characteristics of these patients, hence our inability to conduct a comparative analysis with respondents to determine whether the socioeconomic or health status of these two populations were significantly different. Nevertheless, the questionnaire was presented in the same manner to eligible patients prior to and during/since 2014, each patient being informed that the questionnaire would be systematically analysed by a specialised occupational practitioner. Each patient was also informed that, in the event of the discovery that occupational exposure was likely linked to their disease, they would be advised on ways to claim for occupational recognition. Since the purpose of our study is not to analyse questionnaire performance but to compare the number of OD claims among respondents before and since 2014, we believe that this limit does not does not significantly affect our results.

Even if certain professions are likely to be exposed to several carcinogenic substances, we did not collect detailed data on all the professions of the exposed patients (specific profession, tasks for each profession, duration of exposure, etc.). Indeed, in France, it is only possible to claim for OD recognition on the basis of one carcinogenic agent. This is why we exclusively reported the agent retained by the occupational physician as the most pertinent for a claim. It would have been interesting to collect further information on the most frequently represented trades or professional categories. The exclusive focus on a single most relevant carcinogen per patient in the study underestimates the presence of multi-exposure in occupational settings.

Furthermore, after analysing the questionnaires, particularly in the search for a link between LC and occupational exposure, the exposure of 1229 (61%) patients was not sufficiently characterised to be eligible to claim for OD recognition. Questions regarding the possibility of remotely questioning these patients once more remain, since information on occupational exposure may be lacking, and patients may have been suffering from concentration or memory disorders when completing the questionnaire. Certain patients may also underestimate occupational exposure due to their perception of LC being caused by smoking.

The structure and diversity of data were not conducive to a relevant clustering classification. However, we believe these two approaches are particularly interesting to refine and enrich our results in the context of a future protocol that would include qualitative data.

Our study is nevertheless original, and the 14-year analysis period allowed us to confirm the importance of occupational exposure associated with LC, the pertinence of occupational questionnaires to improve the detection of occupational exposures, and the role of specialised OD consultations in facilitating compensation for patients presenting with LC. Our study is the only one, to our knowledge, that has precisely evaluated the value of a specialised ‘face-to-face’ OD consultation in addition to the unique use of a questionnaire (studies found in the scientific literature did not evaluate the specific contribution of specialised OD consultation in helping patients with LC obtain compensation and in reducing under-recognition).

These data show that involving occupational specialists through dedicated consultations can dramatically reduce the under-recognition of work-related LC. In practice, this means questioning every LC patient on their professional career, issuing exposure questionnaires, and referring suspicious cases to specialised OD consultations.

## 6. Conclusions

Our study demonstrates the importance of a physician trained in OD within a university hospital in reducing the underreporting of occupational LC and in helping patients to claim for OD recognition. Specialised OD consultations have demonstrated their importance within a context where general practitioners, pneumologists and oncologists have insufficient time to inform patients of their ability to claim for OD recognition of their cancer. Our results highlight the importance of continuing and generalising specialised OD consultations to other healthcare establishments, contributing to countering the under-reporting of cases of occupational LC. We wish to continue our study by integrating patients assessed in consultations for cancers of the bladder, kidney and for malignant haemopathies. We would like to use semi-directed interviews or open questionnaires in order to provide a source for root cause analysis via an Ishikawa diagram, while a more comprehensive database could allow for segmentation by clustering. Given the high rate of refusal and abandoned claims, further information and the possibility of the most vulnerable patients benefitting from support for their recognition claim are elements that warrant consideration to limit social inequalities.

## Figures and Tables

**Table 1:** Study patient characteristics.

	Total Population (N = 621)
Sex	
Men: n (%)	608 (97.9%)
Women: n (%)	13 (2.1%)
Mean age at completion of the questionnaire (years)	64 (±8)
Carcinogenic agent: n (%)	
Asbestos	521 (84%)
Silica	17 (2.7%)
Welding fumes	17 (2.7%)
Chromium	5 (0.8%)
Iron	5 (0.8%)
Coal tar	9 (1.4%)
Polycyclic aromatic hydrocarbons from petroleum derivatives	4 (0.6%)
Polycyclic aromatic hydrocarbons from coal combustion soot	3 (0.5%)
Polycyclic aromatic hydrocarbons from cutting oils	3 (0.5%)
Diesel engine fumes	16 (2.6%)
Painting trade	15 (2.4%)
Passive smoking	3 (0.5%)
Ionising radiation	2 (0.3%)
Cobalt dust	1 (0.2%)
Claim filed for occupation disease recognition	
Yes	202 (32.6%)
No	419 (67.4%)
Specialised occupational disease consultation	
Yes	229 (36.9%)
No	392 (63.1%)
Smoking status	
Non-smoker	22 (3.6%)
Former smoker (smokefree)	410 (66%)
Active smoker	178 (28.7%)
Unknown	11 (1.8%)
Histology	
Adenocarcinoma	326 (52.5%)
Epidermoid carcinoma	168 (27.0%)
Large cell undifferentiated carcinoma	45 (7.3%)
Adenosquamous carcinoma	7 (1.1%)
Sarcomatoid carcinoma	2 (0.3%)
Neuro-endocrine tumour	57 (9.2%)
Typical or atypical carcinoid tumour	10 (1.6%)
Other (pleiomorphic, small cell anaplasic)	2 (0.3%)
Unknown	4 (0.7%)

**Table 2:** Comparison of patients included in groups 1 and 2 (univariate analysis).

	Group 1 (Patients Without Specialised Consultation)	Group 2 (Patients with Specialised Consultation)	*p*-Value (Group 1/Group 2)
	N = 392	N = 229	
Sex			
Men: n (%)	385 (98.2%)	223 (97.4%)	0.564
Women: n (%)	7 (1.8%)	6 (2.6%)	
Age at study (years)	63 (±9)	65 (±7)	0.00038
Carcinogenic agent from MPI * table: n (%)			NA
Chrome	4 (1.0%)	2 (0.9%)	
Coal tar	9 (2.3%)	3 (1.3%)	
Crystalline silica	14 (3.6%)	1 (0.4%)	
Asbestos	309 (78.8%)	212 (92.6%)	
Radon in iron ore mines	5 (1.3%)	0 (0)	
Ionising radiation	0 (0)	1 (0.4%)	
Cobalt	0 (0)	1 (0.4%)	
Other (welding fumes, diesel engine fumes, hydrocarbons, painting trade)	51 (13.0%)	9 (4.0%)	
Claim filed for occupational disease recognition			
Yes	45 (11.5)	157 (68.5)	2.87.10^−48^
No	347 (88.5)	73 (31.9)	
Smoking status			
Non-smoker	10 (2.5%)	13 (5.7%)	8.9.10^−14^
Former smoker (smokefree)	222 (56.7%)	187 (81.6%)	
Active smoker	148 (37.7%)	29 (12.7%)	
Unknown	12 (3.1%)	0 (0)	
Histology			NA **
Adenocarcinoma	194 (49.4%)	132 (57.6%)	
Epidermoid carcinoma	112 (28.6%)	56 (24.5%)	
Large cell undifferentiated carcinoma	35 (9.0%)	10 (4.4%)	
Adenosquamous carcinoma	4 (1.0%)	3 (1.3%)	
Sarcomatoid carcinoma	2 (0.5%)	0 (0)	
Neuro-endocrine tumour	35 (9.0%)	22 (9.6%)	
Typical or atypical carcinoid tumour	4 (1.0%)	6 (2.6%)	
Other (pleiomorphic, small cell anaplasic)	2 (0.5%)	0 (0)	
Unknown	4 (1.0%)	0 (0)	

* ‘Maladie Professionnelle Indemnisable’ (Occupational disease eligible for compensation). ** Not applicable.

**Table 3:** Patients with specialised occupational disease consultation according to sex, age, claim filed for OD recognition and smoking status (univariate and multivariate analysis).

	Univariate Model		Multivariate Model	
	OR [95% CI]	*p*-Value	OR [95% CI]	*p*-Value
Sex Women vs. Men	1.43 [0.48–4.32]	0.360	-	
Age at study (years)	1.03 [1.01–1.05]	0.034	1.05 [1.02–1.07]	<0.001
Claim filed for ODR ^1^ Yes vs. No	16.32 [10.73–24.82]	<0.001	18.13 [11.47–28.64]	<0.001
Smoking status		<0.001		<0.001
Non-smoker vs. Active smoker	6.63 [2.66–16.57]	<0.001	4.90 [1.54–15.53]	0.007
Former smoker vs. Active smoker	4.30 [2.76–6.70]	<0.001	3.08 [1.79–5.28]	<0.001

^1^ ODR: Occupational Disease Recognition.

**Table 4:** Comparison between patients having filed or not filed a claim for occupational disease recognition (univariate analysis).

	Subjects Who Did Not File an Occupational Disease Recognition Claim	Subjects Who Filed an Occupational Disease Recognition Claim	*p*-Value
Number of subjects	419	202	
Sex			
Men (n%)	413 (98.6%)	195 (96.5%)	0.132
Women (n%)	6 (1.4%)	7 (3.5%)	
Mean age at time of claim (years)	64 (±9)	63 (±8)	0.34
Carcinogenic agent: n (%)			NA *
Chromium	3 (0.7%)	3 (1.5%)	
Coal tar	9 (2.2%)	3 (1.5%)	
Crystaline silica	12 (2.9%)	3 (1.5%)	
Asbestos	341 (81.3%)	180 (89.1%)	
Radon in iron ore mines	4 (1.0%)	1 (0.5%)	
Ionising radiation	1 (0.2%)	0 (0)	
Cobalt	0 (0)	1 (0.5%)	
Other (welding fumes, diesel engine fumes, hydrocarbons, painting trade)	49 (11.7%)	11 (5.4%)	
Specialised occupational disease consultation			*p* < 0.01
Yes before 2014	5 (1.2%)	6 (3.0%)	
Yes since 2014	67 (16.0%)	151 (74.7%)	
No before 2014	289 (69.0%)	28 (13.9%)	
No since 2014	58 (13.8%)	17 (8.4%)	
Smoking status			
Non-smoker	13 (3.1%)	10 (5.0%)	2.8.10^−5^
Former smoker (smokefree)	254 (60.6%)	155 (76.7%)	
Active smoker	142 (33.9%)	35 (17.3%)	
Unknown	10 (2.4%)	2 (1.0%)	
Histology			NA *
Adenocarcinoma	203 (48.4%)	123 (60.9%)	
Epidermoid carcinoma	124 (29.6%)	44 (21.8%)	
Large cell undifferentiated carcinoma	37 (8.8%)	8 (3.9%)	
Adenosquamous carcinoma	4 (1.0%)	3 (1,5%)	
Sarcomatoid carcinoma	2 (0.5%)	0 (0)	
Neuro-endocrine tumour	37 (8.8%)	20 (9.9%)	
Typical or atypical carcinoid tumour	7 (1.7%)	3 (1.5%)	
Other (pleiomorphic, small cell anaplasic)	2 (0.5%)	0 (0)	
Unknown	3 (0.7%)	1 (0.5%)	

* Not applicable.

## Data Availability

All data generated or analysed during this study are included in this published article [and its Supplementary Information Files]. The datasets used and/or analysed during the current study are available from the corresponding author upon reasonable request.

## Supplementary Materials

The following supporting information can be downloaded at: https://www.mdpi.com/article/10.3390/ijerph22060927/s1, Occupational questionnaire.

## Abbreviations

LC	lung Cancer
OD	occupational Disease
CI	confidence interval
OR	odds ratio
IARC	international agency for research on cancer
SD	standard deviation
