# Supplementary material for: Compensation for Patients with Work-Related Lung Cancers: Value of Specialised Occupational Disease Consultations to Reduce Under-Recognition
Source: Int J Environ Res Public Health. 2025 Jun 12;22(6):927. doi: 10.3390/ijerph22060927 (PMC12193440; doi:10.3390/ijerph22060927)
Supplement: Supplementary file 1 [file ijerph-22-00927-s001.zip › ijerph-3581807-supplementary.pdf]

OCCUPATIONAL QUESTIONNAIRE

Patient number: □ □□□

Gender: Male Female

Date of birth: _ _ / _ _ /_ _

National insurance number: _ _ _ _ _ _ _ _ _ _ _ _ _ _ _ _

Date :-----------------------

MEDICAL INFORMATION

Diagnosis:

- Localisation:-----------------------------------
- Date: ---------------------------
- Anatomopathology:

  Epidermoid Small cell. Adenocarcinoma

  Large cell Adenosquamous

  Other Please specify: --
- Classification T ---- N ---- M ----

Presence of:

- Asbestosis on histology Yes No Unk.
- Pleural fibrosis on histology Yes No Unk.
- Pleural fibrosis on TDM Yes No Unk.
  (over and above tumour zone thickening)
- Silicosis on TDM Yes No Unk.
- Silicosis on histology Yes No Unk.

TOBACCO

- **Are you:**

  Non-smoker

  Active smoker

  Former smoker (smokefree for at least one year)

- **Duration (if applicable)**

  Start Year: _ _ _ _

  End year: _ _ _ _

- **Cigarettes** (number per day):
  - from ---- to ----: ----------/day
  - from ---- to ----: ----------/day
  - from ---- to ----: ----------/day
  - from ---- to ----: ----------/day

- **Cigars** (number per day)**:**
  - from ---- to ----: ----------/day
  - from ---- to ----: ----------/day
  - from ---- to ----: ----------/day
  - from ---- to ----: ----------/day

- **Pipe** (package weight in grammes and number of packages per month):**----------------g**
  - from ---- to ----: ----------/month
  - from ---- to ----: ----------/month
  - from ---- to ----: ----------/month
  - from ---- to ----: ----------/month

Total duration of smoking (in years)...

Cumulative smoking (packs-years)...

EDUCATION AND VOCATIONAL TRAINING

- **Father's occupation ------------------------**
- **Mother's occupation ------------------------**
- **Spouse's occupation ------------------------**

- **At what age did you leave compulsory schooling? ------------------------**

What is your level of education?---

- **Have you followed one or more professional training courses?**

yes **no**

If yes, please specify:

Training dates: Level passed:

From --------- to ----------Level: -----------------------------------

From --------- to ----------Level: -----------------------------------

From --------- to ----------Level: -----------------------------------

From --------- to ----------Level: -----------------------------------

(Please complete a professional activity page for each period)

- **Have you ever served in a military position?**

yes **no**

Please specify the period: from ---------- to ----------

(Do not forget to complete a professional activity page for military service).

PROFESSIONAL ACTIVITY

Year started: _ _ _ _ **Year ended:** _ _ _ _

Name and address of employer: ---

Employer's main activity: ---

Employer's secondary activities: ---

Approximate number of employees:

10 - 49 50 – 199 > 200

Your position in the organisation: ---

Working hours (indicate with a circle)**:**

- **Part-time: ------------------ (hours/week)**
- **Full-time**

Job description (type of premises, specific tasks and time worked for each task, machines, processes and products or materials used, protective measures):

Names of products used (brands): ---

At your workstation, was there (circle if applicable and specify type)**?**

- dust: ---
- fumes: --
- odours: --
- steam: ---

Individual and collective protection (circle if applicable):

- cabin, hood, ventilation, suction
- goggles
- gloves
- masks
- clothing
- other (please specify):---

What tasks were carried out by colleagues around your workstation?

Have you already stood in for a colleague? If yes, for what task and for how long:

Time:---

National insurance scheme covering this work period: --

General national insurance scheme	Hospital-based civil service
Agricultural insurance scheme	Special schemes
Civil service, French State	Self-employed workers' scheme
Territorial civil service	Other. Please specify:________________________

Expertise:

	Prob	Freq	Int
Asbestos	☐	☐	☐
Silica	☐	☐	☐
PAH	☐	☐	☐
Diesel	☐	☐	☐
Be	☐	☐	☐
Cr	☐	☐	☐
Ni	☐	☐	☐
CoW	☐	☐	☐
Cd	☐	☐	☐
BCME	☐	☐	☐
As	☐	☐	☐
Gas/ dust radioact.	☐	☐	☐
iron ore	☐	☐	☐
Paint	☐	☐	☐

Probability:
- 0 not exposed
- 1 passive expo.
- 2 poss direct expo.
- 3 prob /confirmed. expo.

Frequency:
- 1 < 1 /month
- 2 1/m > and < 50% time
- 3 > 50 % time

COMPLEMENTARY OCCUPATIONAL QUESTIONNAIRE

- Did you use protective measures against heat (gloves, protective clothing, plates, cushions, etc.)?

Yes No Unk.

If yes, Which: --

From _ _ _ _ to _ _ _ _

Frequency: --

- Have you carried out welding work?

Yes No Unk.

If yes, Type of welding: ---

From _ _ _ _ to _ _ _ _

Frequency: --

Have you handled asbestos joints or any other material containing asbestos?

Yes No Unk.

If yes, Which material: --

From _ _ _ _ to _ _ _ _

Frequency: --

Have you carried out insultation work or boiler maintenance?

Yes No Unk.

If yes, Type of work: --

From _ _ _ _ to _ _ _ _

Frequency: --

- Have you carried out maintenance or replacement work on vehicle brakes or clutch systems?

Yes No Unk.

If yes, Type of work: --

From _ _ _ _ to _ _ _ _

Frequency: --

- Have you carried out painting, plastering or priming work?

Yes No Unk.

If yes, Type of work: --

From _ _ _ _ to _ _ _ _

Frequency: --

- Have you carried out demolition work?

Yes No Unk.

  - If yes, Type of work: --
  - From _ _ _ _ to _ _ _ _
  - Frequency: ---

- Have you been in contact with artificial mineral fibres (glass fibre, rock fibre, slag fibre, ceramic fibre)?

Yes No Unk.

If yes, Type of fibre: --

From _ _ _ _ to _ _ _ _

Frequency: ---

- Have you been in contact with other fibres?

Yes No Unk.

If yes, Type of fibre: --

From _ _ _ _ to _ _ _ _

Frequency: ---

- Have you been in contact with crystalline silica (glass industry, use of sand and quartz, ceramic industry, earthwork, etc.)?

Yes No Unk.

If yes, Circumstances: --

From _ _ _ _ to _ _ _ _

Frequency: ---

- Have you handled tar, bitumen, coal and its byproducts?

Yes No Unk.

If yes, Type of work: --

From _ _ _ _ to _ _ _ _

Frequency: ---

- Have you handled used or recycled mineral oils?

Yes No Unk.

If yes, Circumstances: --

From _ _ _ _ to _ _ _ _

Frequency: ---

- Have you handled beryllium (alloy, jewellery)?

Yes No Unk.

If yes, Circumstances: --

From _ _ _ _ to _ _ _ _

Frequency: --

- Have you worked in the iron or aluminium industry?

Yes No Unk.

If yes, Position: --

From _ _ _ _ to _ _ _ _

Frequency: --

- Have you been in contact with chromium (chrome plating, painting, colorants, alloys)?

Yes No Unk.

If yes, Type of work: --

From _ _ _ _ to _ _ _ _

Frequency: --

- Have you carried out nickel plating and/or worked in nickel extraction or transformation?

Yes No Unk.

If yes, Type of work: --

From _ _ _ _ to _ _ _ _

Frequency: --

- Have you worked on the production and/or transformation of polyvinyl chloride (PVC)?

Yes No Unk.

If yes, Type of work: --

From _ _ _ _ to _ _ _ _

Frequency: --

- Have you sprayed crops with products containing arsenic?

Yes No Unk.

If yes, Circumstances: --

From _ _ _ _ to _ _ _ _

Frequency: --

- Have you been in contact with radioactive products and/or ionising radiation?

Yes No Unk.

If yes, Circumstances: --

From _ _ _ _ to _ _ _ _

Frequency: --

- Have you used strong acids to etch/strip surfaces?

Yes No Unk.

If yes, Circumstances: --

From _ _ _ _ to _ _ _ _

Frequency: --

- Have you carried out any of the aforementioned activities outside of your professional environment?

Yes No Unk.

If yes, which? Please specify years and frequencies:

CONCLUSION

- Agents retained within the framework of the General Social Security scheme criteria:
  - Ionising radiation (6) | Chromates (10ter) | PAH (16bis)

Ionising radiation (6)	Chromates (10ter)	PAH (16bis)
Arsenic (20bis)	Gold arsenic (20ter)	Silica (25 A)
Asbestos (30 C)	Asbestos (30bis)	Nickel (37ter)
Iron oxide (44bis)	CoW (70ter)	BCME (81)

  - Not listed. Please specify:---

- Occupational disease claim proposed?

  Yes No Unk.

  - If no, grounds for refusal:

    Refusal by patient or successors

    Alteration in general state of health or death

    Exposure in a profession that does not allow oforccupational disease recognition

    Other:________________________________

  - If yes, date of medical certificate: _ _ / _ _ / _ _ Unk.

- FIVA compensation claim filed:

  Yes No Unk.
